# Recognizing acute delirium as part of your routine [RADAR]: a validation study

**DOI:** 10.1186/s12912-015-0070-1

**Published:** 2015-04-01

**Authors:** Philippe Voyer, Nathalie Champoux, Johanne Desrosiers, Philippe Landreville, Jane McCusker, Johanne Monette, Maryse Savoie, Sylvie Richard, Pierre-Hugues Carmichael

**Affiliations:** 1 Faculty of Nursing Sciences, Laval University, Pavillon Ferdinand-Vandry, room 3445, 1050, rue de la Médecine, Quebec City, Quebec Canada; 2 Centre d’excellence sur le vieillissement de Québec, Quebec City, Quebec Canada; 3 Institut Universitaire de Gériatrie de Montréal, Département de médecine familiale, Montreal University, Montreal, Quebec Canada; 4 Faculty of Medicine and Health Sciences, Sherbrooke University, Sherbrooke, Quebec Canada; 5 École de psychologie, Laval University, Quebec City, Quebec Canada; 6 St. Mary’s Research Centre, St Mary’s Hospital, Montreal, Quebec Canada; 7 Department of Epidemiology, Biostatistics and Occupational Health, McGill University, Montreal, Quebec Canada; 8 Division of Geriatric Medicine, Jewish General Hospital and Donald Berman Maimonides Geriatric Center, Montreal, Quebec Canada; 9 Ste. Anne Hospital, McGill University, Montreal, Quebec Canada

**Keywords:** Delirium, Screening tool, Elderly, Long-term care, Acute care, Cognitive impairment

## Abstract

**Background:**

Although detection of delirium using the current tools is excellent in research settings, in routine clinical practice, this is not the case. Together with nursing staff, we developed a screening tool (RADAR) to address certain limitations of existing tools, notably administration time, ease-of-use and generalizability. The purpose of this study was not only to evaluate the validity and reliability of RADAR but also to gauge its acceptability among the nursing staff in two different clinical settings.

**Methods:**

This was a validation study conducted on three units of an acute care hospital (medical, cardiology and coronary care) and five units of a long-term care facility. A total of 142 patients and 51 residents aged 65 and over, with or without dementia, participated in the study and 139 nurses were recruited and trained to use the RADAR tool. Data on each patient/resident was collected over a 12-hour period. The nursing staff and researchers administered RADAR during the scheduled distribution of medication. Researchers used the Confusion Assessment Method to determine the presence of delirium symptoms. Delirium itself was defined as meeting the criteria for DMS-IV-TR delirium. Inter-rater reliability, convergent, and concurrent validity of RADAR were assessed. At study end, 103 (74%) members of the nursing staff completed the RADAR feasibility and acceptability questionnaire.

**Results:**

Percentages of agreement between RADAR items that bedside nurses administered and those research assistants administered varied from 82% to 98%. When compared with DSM-IV-TR criterion-defined delirium, RADAR had a sensitivity of 73% and a specificity of 67%. Participating nursing staff took about seven seconds on average, to complete the tool and it was very well received (≥98%) overall.

**Conclusions:**

The RADAR tool proved to be efficient, reliable, sensitive and very well accepted by nursing staff. Consequently, it becomes an appropriate new option for delirium screening among older adults, with or without cognitive impairment, in both hospitals and nursing homes. Further projects are currently underway to validate the RADAR among middle-aged adults, as well as in newer clinical settings; home care, emergency department, medical intensive care unit, and palliative care.

**Electronic supplementary material:**

The online version of this article (doi:10.1186/s12912-015-0070-1) contains supplementary material, which is available to authorized users.

## Background

Although reliable and valid tools exist to help clinicians recognize the presence of delirium [1-3], 53-75% of delirium cases go undetected in acute care [4-8], 49-87% in long-term care [9,10] and 46% in home care [11]. To date, the Confusion Assessment Method (CAM) is the most widely used instrument for detecting the presence of delirium in both clinical and research settings [1,2,12,13]. The CAM takes five to 10 minutes to administer. It was initially developed for use in internal medicine units and later adapted for use in intensive care units (ICU), emergency and long-term care settings [12,14,15]. It has been translated into 10 languages and several delirium clinical practice guidelines recommend its use [13]. Although high sensitivities (93%-100%) and specificities (98%-100%) were observed in validation studies where research nurses administered the CAM [14], its sensitivity is lower when bedside nurses use it in routine clinical practice (sensitivities and specificities between 19-47% and 98-100% respectively) [4,6,8,16].

Despite the long-established use of the CAM in research, several barriers have hampered efforts to implement the CAM as a screening tool for delirium in clinical settings. Our accumulated research and clinical experience, the criticism of detection tools voiced by bedside nurses, and previously published studies, all led to our decision to tackle the issue of delirium that goes undetected by nurses.

Time constraint is one of the most frequently cited barriers to daily delirium screening [17-20]. Two systematic reviews recommended the use of delirium tools that require an administration time of from five to 10 minutes [1,3]. In Canada, nurses typically care for 10 patients in acute care hospitals and up to 36 residents in nursing homes. Consequently, if nurses were to screen with the CAM all their patients/residents as recommended, they would have to find 50 to 180 unrestricted minutes in one shift, depending on the clinical setting. Obviously, no bedside nurses have this time available. Besides, this is the minimum amount of time required since more than one assessment over a 24-hour period is often necessary to detect a delirium given its fluctuating nature during the course of the day [21]. If the assessment is based on a single screening when the patient is doing well, the delirium will go undetected [22].

Perceived difficulty using the tools is yet another important barrier to delirium screening [17]. Several factors related to the ease of use of scales have been identified; one being the level of expertise required by the rater [1]. For example, some of the available delirium tools are designed to be administered by clinicians with psychiatric experience [23]. Given the scarcity of clinicians with such expertise in many clinical settings, these tools might not be used for systematic screening. The quality of the training provided in the use of the tool affects the level of confidence in performing the assessment and has been reported as an important factor in surveys on nurses’ attitudes toward delirium screening [19]. Moreover, research has shown that inadequate training can seriously compromise the detection accuracy of the tool [13,22]. The length of the training required in order to use the tool properly and the cost involved in such training, is another barrier to take into account. Tools that require long training are costly for an organization’s budget. Another factor that affects screening scale acceptability is how taxing it is for the patient [1]. Nurses were critical of the need for patients to concentrate and cooperate as it limits the number of assessments that can be done [7]. For instance, the questioning and testing required by some scales [24] may demand too much of an already fragile individual and result in the clinicians deciding to cease screening. Finally, the screening process can be further complicated by the need to know the patient’s baseline functioning [24] or to obtain more information on it by consulting other sources (medical chart, staff and family members). Obtaining knowledge about a patient’s usual functioning can present a challenge in settings with high staff or patient turnover (e.g. emergency department) [25] and where such enquiries can be time-consuming.

Other criticism of screening scales relates more to their generalizability and validity. The generalizability of a scale is important. The existing tools were developed for use in specific settings (ICU, emergency, internal medicine, etc.) or among a particular population (cancer patients) [26].Therefore they may not be suitable for systematic screening for delirium in acute and long-term care settings. Besides, some scales have not been validated for individuals with dementia [27]. This can be problematic, given that dementia is a leading risk factor for delirium [28] and its prevalence in patients admitted to internal medicine units can reach 42% [29]. Validity refers to the extent to which a tool accurately measures the underlying construct that it is intended to measure. While certain available instruments, such as the CAM, are based on the Diagnostic and Statistical Manual of Mental Disorders (DSM) criteria for delirium, others measure a broader concept of confusion rather than criterion-defined delirium, and so their usefulness is limited [3,30]. Repeated assessment of delirium over a short period could also introduce a bias and affect the internal validity of the scale [31]. For example, if the delirium screening tool requires the patients to do a certain task [32], the patients may improve with repetition and not because of an actual change in functioning. Conversely, fatigue may set in after repeated testing, potentially altering patient performance and leading to false conclusions.

Our research group joined forces with nurses to identify strategies for overcoming the barriers to delirium screening. To address time constraints and the need to do many delirium screenings daily, the tool had to be brief (less than one minute). In addition, since our goal was to limit any additional workload for nurses, the screening had to be integrated into activities already part of nurses’ routines. We also identified several key screening tool characteristics to optimize ease of use. To make best use of staff resources, the required level of rater expertise could not be restricted to just a few staff members. For this reason we determined that licenced practical nurses (LPN), as well as registered nurses (RN), should take part in the screening process [LPN have two years of training and work under the supervision of RN]. To foster the raters’ confidence in their use of the tool, we compiled a comprehensive training package to be included with the tool, and stipulated that training should take less than 45 minutes. To lessen the burden on patients, no direct questioning or testing would be necessary. As mentioned earlier, this also reduces the risk of introducing bias due to repeated testing. To facilitate the screening process, we avoided any need for the rater to know the patient’s usual functioning or to obtain information from other sources. Finally, the screening tool had to be based on delirium criteria (i.e., the DSM-IV-TR) and also be valid among the older adults, with or without the cognitive impairment, typically found in acute and long-term care settings. We kept all these considerations in mind at every stage in the development of RADAR.

The RADAR (*Repérage Actif du Delirium Adapté à la Routine* or Recognizing Acute Delirium As part of your Routine) is a screening tool designed to be administered by nursing staff (LPN and RN) during the scheduled distribution of medications. Since staff give patients medication many times over a 24 hour period, RADAR offers plenty of opportunities for delirium screening. The tool contains three simple observation-based items (yes-no) that are intended to identify the presence of signs of a possible delirium. Completion of the RADAR is based solely on observation of the patient by a member of the nursing staff during their interaction at the scheduled distribution of medication. The patient is not questioned directly, there is no need to seek additional information from other sources (e.g. medical chart, family, etc.) and no need to know the patient’s usual functioning. In this paper, we present the results of a large scale study to validate the final version of RADAR (Additional file 1) among older patients/residents in acute and long-term care settings. The following reliability and validation tests will be examined: inter-rater agreement, convergent validity and concurrent validity. Finally, staff perceptions about the RADAR will also be evaluated. Additional file 2 describes the earlier steps that led to this version.

## Methods

### Design, settings and selection of participants

This validation study was conducted on three units (medical, cardiology and coronary care) of an acute care hospital (ACH) and five units of a long-term care (LTC) facility. Data collection took place from November 2012 to November 2013. The Research Ethics Boards of each participating facility approved the study: Université Laval, CHU de Québec, and Centre de santé et des services sociaux de la Vieille-Capitale.

#### Patients/residents enrolment

Patients (ACH)/residents (LTC) with, or without a medical diagnosis of dementia in their medical chart were included in the study if they were aged 65 years or over and were given medication on at least two occasions during the day. We excluded anyone with a history of psychiatric illness (specifically psychotic disorders, bipolar disorders and major depressive disorders with psychotic features) and those with intellectual disabilities. A research assistant compiled weekly lists of all eligible patients/residents for each study site. There were different recruitment procedures for individuals with and without cognitive impairment. The presence of cognitive impairment was defined as the presence of any indicators (e.g. dementia, confusion, disorientation, etc.) in the medical chart or reported by the bedside nurse, which could affect the patient/resident’s ability to make an informed decision regarding study participation. For individuals with cognitive impairment, institution staff sent an informative letter about the study to the legal guardians or, in the absence of such, to the family member designated as guardian, and invited them to contact the research assistant for further information. This letter was sent to all families. Respondents who agreed to participation on behalf of another were requested to sign proxy consent forms. Each week patients/residents for whom consent was obtained were randomly selected to participate in the study. Direct assent to participation was obtained from individuals deemed to have mild or moderate cognitive impairment (according to the research assistant’s clinical judgment). Potential participants from among patients/residents without cognitive impairment were also randomly selected from the weekly list of all those eligible for the study. Their bedside nurse asked them if they would agree to meet with the research assistant to learn about the project. If then they agreed to take part, they were invited to sign the consent form. Up to six patients/residents were recruited each week.

#### Nursing staff enrolment

Part-time and full-time RNs, LPNs and CPNPs (candidates for the profession of nursing practice) who were working day and evening shifts with at least two months work experience in the selected facility and a minimum of five days on the targeted unit in the previous month were invited to take part in the study. We then sought their informed consent.

### Overview of data collection and measures

Researchers gathered data for each patient/resident over a 12-hour period that took in all potential distributions of medication (08:00, 12:00, 17:00 and HS [at bedtime]). This project involved two research assistants (RAs) per study site. These RAs were one research assistant with a Master’s degree in Nursing Sciences and another research assistant with a Master’s degree in Experimental Medicine. The first RA, called RA-Delirium, was responsible for using the CAM to determine the presence of delirium and the Hierarchic Dementia Scale (HDS) to assess severity of cognitive impairment. To complete the CAM, RA-Delirium used three data sources (chart, family members, if available, and nursing staff) and before completing the CAM, had to make three structured assessments of delirium symptoms over a seven-hour observation period. The second RA, called RA-RADAR, was blinded to the CAM delirium status of patients/residents and to obtain data for inter-rater reliability testing, also administered RADAR, at exactly the same time as the bedside nurse. In addition, RA-RADAR measured the time bedside nurses took to complete the RADAR and collected the participant’s baseline characteristics (age, sex, date of admission, level of functional autonomy, level of comorbidity and type of dementia, if any). The completed RADAR was inserted in the folder containing the medication administration record for each participating patient/resident. Participating nursing staff was instructed to complete RADAR during every scheduled distribution of medication.

At the end of the study, RA-Delirium was also to administer a questionnaire to the participating nursing staff in order to rate their perception of RADAR.

### Instruments

#### Primary measures

The RADAR scale used in this validation study consists of three items: “*When you gave the patient his/her medication…*1) *Was the patient drowsy?*; 2) *Did the patient have trouble following your instructions?*; *3) Were the patient’s movements slowed down?* A RADAR screening is considered positive when at least one item is checked “Yes”.

The presence of delirium symptoms was measured with the CAM, an established and widely used instrument to help in the detection of delirium in both clinical and research settings [12]. This instrument, which takes 10 minutes to complete, was designed to allow non-psychiatrist clinician to capture the features of delirium specified in the revised third edition of the Diagnostic and Statistical Manual of Mental Disorders (similar to DSM-IV-TR criteria). In this study, delirium was defined as meeting the criteria for DSM-IV-TR delirium (acute onset, fluctuation over the course of the day, altered level of consciousness with inattention and at least one of the following symptoms: memory impairment, disorientation, disorganized speech and perceptual disturbances). The CAM has been shown to be sensitive (94% to 100%) and specific (90% to 95%) compared to the diagnosis of a psychiatrist [12,13]. Following procedures similar to what we used in earlier studies on delirium [9,33], RAs were trained to recognize the symptoms of delirium using the CAM. This involved clinical demonstrations, trial ratings of cases and discussion of findings until such time as there was satisfactory agreement between the research staff and the principal investigator (PV).

During the course of the study, the RAs assessed a sample of patients (n = 37/193; 19% of total sample) simultaneously and independently to check inter-rater reliability on the CAM. Percentage of agreement on each item of the CAM varied from 72% to 100%, while kappa values ranged from 0.30 to 1.

In this validation study the RADAR feasibility and acceptability questionnaire, developed by the research team for Steps 3 and 4 (Additional file 2), was used to collect data on the nursing staff’s perceptions of several aspects of RADAR (e.g., ease of use, clarity of items, training required). In addition, it was used to obtain input on strategies to further improve the scale so as to facilitate its implementation in clinical settings.

#### Instruments for descriptive measures

Cognitive impairment severity was assessed by the Hierarchic Dementia Scale (HDS) [34]. Maximum score for the entire scale is 200 points. Older adults who are cognitively intact generally score the maximum number of points, or close to it [35]. This scale displays almost no floor effect i.e., participants with severe dementia can respond correctly to some of the easiest items and obtain scores other than zero [36]. The HDS has well established validity and reliability [35]. For example, Ronnberg and Ericsson [36] reported a test-retest reliability of 0.96 (Spearman rho coefficients) and a concurrent validity coefficient of 0.86 between the HDS and the Mini Mental Status Examination. More recently, Engelborghs and colleagues [37] demonstrated a significant correlation between HDS scores and biological markers of Alzheimer’s disease.

Functional autonomy was measured according to the Functional Autonomy Measurement System (SMAF) [38]. This 29-item scale measures functional ability in five areas: activities of daily living (ADL: seven items), mobility (six items), communication (three items), mental functions (five items) and instrumental activities of daily living (IADL: eight items). In this study, we used a modified 20-item version for institutionalized participants (excluding the eight IADL items and one item related to exterior mobility). The disability for each item is scored on a 5-point scale: 0 = independent, 0.5 = with difficulty, 1 = with supervision, 2 = with help and 3 = dependent. This version of the scale has shown good test-retest and inter-rater reliability (ICC of 0.95 and 0.96 respectively) [39]. Higher score is indicative of severe autonomy impairment.

Data extracted from the participants’ medical charts included: demographic information (e.g. age, sex, and years since admission), diagnosis of dementia, and other medical diagnoses. Information on medical problems was used to compute the Charlson Comorbidity Index [40], validated as a predictive index for survival among older patients and nursing home residents [41,42]. The overall score ranges from 0 to 37, with higher scores indicating greater comorbidity.

### Training nursing staff on the use of RADAR

Training consisted of a 15-minute PowerPoint presentation on delirium, its manifestations as well as instructions on how to administer and score the RADAR items. This presentation included four one-minute videos showing simulated nurse-patient interactions during the medication distribution process. Following each viewing, and to ensure they correctly understood the information, all participants had to complete the RADAR. In addition, the RA gave at least two individual coaching sessions to the participating nurses (RN and LPN). One was conducted at the patient’s bedside during the first completion of RADAR by the participating nurse. It consisted of reviewing the completed items for validation. The second individual coaching session took place every four weeks, or as required, and involved reviewing the instruction on how to complete each RADAR item.

### Statistical analyses

As previously stated, a RADAR screening was deemed positive if at least one of the three items was checked as present. Participants were said to be positive for RADAR if they received at least one positive RADAR in the day. RADAR screenings administered by either the RA-RADAR or the nurse participant were considered in the analyses. As expected, simultaneous administration of RADAR during the day (by RA-RADAR and nurse) was not always feasible. Sometimes, and for various reasons, only the RA administered the RADAR; at other times only the nurse participant. For instance, the RADAR might be in the folder of a patient under the care of a nurse who did not, or was not eligible to participate in the study. Alternatively, the RA-RADAR might have been busy observing another participant on a different unit and therefore, was unavailable. It is also important to note that, because RA-RADAR was not present during night and evening shifts, only nurse-administered RADAR assessments were available for this period (distribution at 17:00 and HS).

For each RADAR item, we evaluated inter-rater reliability between the item administered by the bedside nurse and that administered by the RA-RADAR (simultaneous assessments were conducted in more than 50% of all RADAR administrations). We then quantified the inter-rater agreement using the kappa statistic in conjunction with raw agreement percentages.

Second, the convergent validity between the RADAR items and their corresponding CAM delirium symptom, as measured by RA-Delirium was evaluated. We computed the proportion of agreement (percent agreement) and kappa values with their confidence intervals. Third, using a DSM-IV-TR criterion-defined delirium as gold standard, we used sensitivities (SE), specificities (SP), positive and negative predictive values (PPV and NPV respectively) and their confidence intervals and calculated the concurrent validity of each RADAR item and the RADAR scale as a whole.

To present the participants’ characteristics and the nursing staff’s perception concerning the use of RADAR in their clinical practice, researchers used means and standard deviations or frequencies and percentages to describe continuous and discrete variables respectively. All analyses were carried out using SAS for Windows, version 9.3.

## Results

### Description of the study population

**Figure 1:**
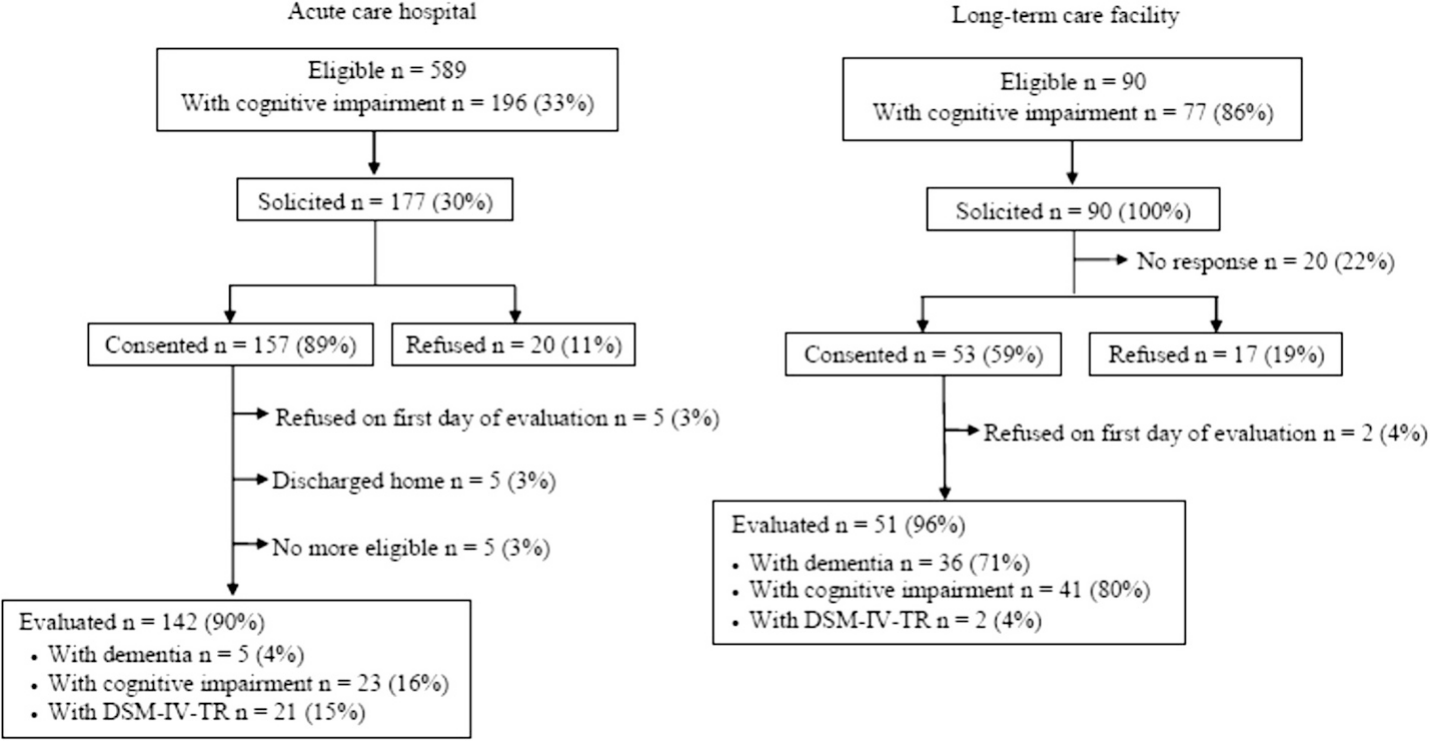
**Flow chart.**

A total of 589 patients and 90 residents met the eligibility criteria (Figure 1). Of these, 267 (38%) were solicited to participate in the study and 210 (79%) consented. Of those who consented, 17 (7%) were not evaluated (seven revoked their consent, five were discharged home and five others were no longer eligible), leaving a sample for analysis consisting of 142 (74%) patients and 51 (26%) residents. Table 1 shows the characteristics of these patients/residents. Mean age was 81 years, 37 (19%) had a high level of comorbidity (score ≥8 on the *Charlson Comorbidity Index*) and 24 (12%) showed substantial deterioration in their functional autonomy (score ≥ 29 on the SMAF). For example, among this latter group, 15 (63%) were completely dependent for personal hygiene, 17 (75%) were dependent for dressing and 15 (63%) were unable to walk. The medical charts of 41 (21%) contained a diagnosis of dementia. A total of 23 (12%) participants met the DSM-IV-TR criteria for delirium.

**Table 1:** **Characteristics of patients/residents**

	Total (N = 193)		Patients = 142 (73.6%)		Residents = 51 (26.4%)	
Variables [Missing]	N (%)	M (SD)	N (%)	M (SD)	N (%)	M (SD)
Age (yrs.)		80.8 (7.8)		79.1 (7.3)		85.5 (7.5)
Sex (female)	116 (60.1)		76 (53.5)		40 (78.4)	
Diagnosis of dementia (yes)	41 (21.2)		5 (3.5)		36 (70.6)	
Types of dementia						
Not specified	3 (7.3)		1 (20.0)		2 (5.6)	
Alzheimer	12 (29.3)		1 (20.0)		11 (30.6)	
Vascular	4 (9.8)		2 (40,0)		2 (5.6)	
Mixed	19 (46.3)		1 (20.0)		18 (50.0)	
Korsakoff	3 (7.3)				3 (8.3)	
DSM-IV-TR delirium	23 (12)		21 (14.8)		2 (3.9)	
Severity of cognitive impairment (HDS) [1]		162.4 (50.9)		178.6 (32.0)		117.5 (65.0)
Mild (≥160)	142 (74.0)		123 (87.2)		19 (37.3)	
Moderate (between 40 and 160)	39 (20.3)		16 (11.4)		23 (45.1)	
Severe (≤40)	11 (5.7)		2 (1.4)		9 (17.7)	
Level of functional autonomy (SMAF)		13.8 (11.6)		9.6 (8.8)		25.4 (10.7)
Mild <29	169 (87.6)		138 (97.2)		31 (60.8)	
Moderate 29-40	19 (9.8)		4 (2.8)		15 (29.4)	
Severe >40	5 (2.6)				5 (9.8)	
Level of comorbidity: (CCI)		6.1 (1.9)		5.8 (1.9)		6.8 (1.9)
Severe ≥ 8	37 (19.2)		21 (14.8)		16 (31.2)	
Number of medications/day		10.1 (3.9)		10.1 (3.8)		10.0 (4.3)
Number of distributions of meds/day		3.6 (0.7)		3.5 (0.7)		3.6 (0.6)

M (SD): Mean (standard deviation); HDS: Hierarchic Dementia Scale; SMAF: Functional Autonomy Measurement System; CCI: Charlson Comorbidity Index.

Descriptive results of the RADAR showed that 64 (33%) participants out of 193 were rated positive for RADAR. Among them, 40 (63%) had one positive RADAR, 16 (25%) had two, six (9%) had three and two (3%) had four RADAR positive out of the four RADAR administrations. Finally, as indicated in Table 2, the proportion of participants rated positive for RADAR increased with the number of daily RADAR administrations received.

**Table 2:** **Proportion of participants rated positive for RADAR according to the number of daily RADAR administrations received**

	Number of daily RADAR administrations			
	1	2	3	4
Total (N = 193)	18	32	53	60
Positive for RADAR (33% n = 64)	2 (11%)	20 (32%)	18 (35%)	24 (40%)

A total of 63 (45%) RN, 70 (50%) LPN and 6 CPNP (4%) consented to take part in the study, representing a recruitment rate of 96%. Mean number of years of experience in nursing and in geriatrics were 10.8 (SD = ±10.3) and 9.7 (SD = ±9.2) respectively. One hundred and three participants (74%) completed the RADAR feasibility and acceptability questionnaire.

### Detailed descriptive data on RADAR

**Figure 2:**
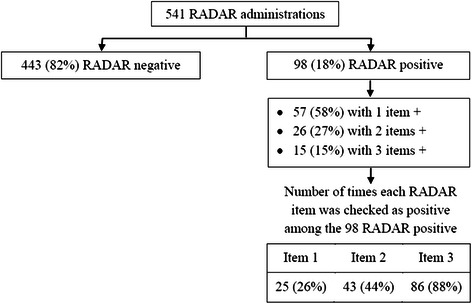
**Descriptive data on RADAR.**

A total of 541 RADAR screenings were administered by bedside nursing staff or RA-RADAR and of those, 98 (18%) were positive (Figure 2). In all the RADAR screenings with a positive score, Item #3 (*Were the patient’s movements slowed down?)* was the most often checked as positive 86 (88%) while Item #1 (*Was the patient drowsy?* was positive 25 (26%) the least often. More than half 57 (58%) of the RADAR screenings with a positive score had one item checked as present, 26 (27%) had two items checked and 15 (15%) had 3.

### Psychometric properties of the 3-item RADAR

#### Inter-rater reliability of RADAR items

The percentages of agreement between the RADAR items administered by the RA and the bedside nurse varied from 82% to 98% (Table 3). Despite this high agreement, the kappa values (0.34 to 0.79) were unstable due to the presence of substantial imbalance in the table’s marginal totals. As presented by Byrt and colleagues [43], this is a manifestation of two paradoxes where, for identical values of agreement, the kappa coefficient can be unduly reduced or increased. It occurs because of a combination of observer bias (when observers disagree on the frequency of the occurrence of a condition) and prevalence bias (when the frequency of the occurrence of a condition is either very low or very high). In our case the prevalence of DSM-IV-TR delirium was rather low (12%).

**Table 3:** **Inter-rater reliability of RADAR items between research assistant and bedside nurse**

RADAR items	Number of inter-rater assessments ^ a) ^	% of agreement	Kappa [95% CI]
Item #1: “*…was the patient drowsy?”*	201/386 (52.1%)	98.0	0.79 [0.59-0.99]
Item #2: “*…trouble following your instructions*?”	201/386 (52.1%)	92.5	0.53 [0.32-0.74]
Item #3: “…*movements slowed down?”*	199/386 (51.6%)	82.4	0.34 [0.18-0.50]

^a)^Radar administration at 08:00 and 12:00 combined.

#### Convergent validity between each RADAR item and the corresponding CAM delirium symptom

The percentages of agreement between the RADAR items and their corresponding CAM symptom ranged from 52% to 85% (Table 4). The lowest percentage of agreement was for RADAR Item #2 “…*did the patient have trouble following your instructions?*” and its corresponding CAM symptom, Inattention. But, this same item also yielded the highest percentage of agreement with Hyper-alert/Agitation. The latter is a combination of the CAM item related to level of consciousness (Item 4 [Hyper-alert]) and Agitation (Item 8). Furthermore, except for this item, percentage of agreement decreases with an increased number of daily CAM assessments on which the CAM item is rated. In other words, when the CAM item is rated based on three daily assessments, percentage of agreement with RADAR is lower than when the CAM item is based on a single daily assessment. It is important to take the number of CAM administrations into account since no clinical settings would ask nursing staff to administer the CAM three times in one shift. Such a request is too demanding time-wise.

**Table 4:** **Convergent validity: Item of RADAR vs. corresponding CAM item**

RADAR items	CAM	Number of CAM assessments on which the CAM item is rated	% of agreement	Kappa [95% CI]
Item #1, “*…was the patient drowsy?”*	CAM 4, Level of consciousness, Hypoalert	1	83.9 (162/193)	0.36 [0.19-0.53]
		2	76.7 (148/193)	0.25 [0.11-0.39]
		3	65.8 (127/193)	0.15 [0.05-0.26]
Item #2, “*…trouble following your instructions*?”	CAM 2, Inattention	1	51.8 (100/193)	0.08 [0.03-0.12]
		2	43.5 (84/193)	0.08 [0.03-0.12]
		3	35.8 (69/193)	0.08 [0.03-0.12]
Item #2, “*…trouble following your instructions*?”	CAM 4 and 8, Hyper-alert/Agitation	1	85 (164/193)	0.16 [−0.02-0.33]
		2	85 (164/193)	0.16 [−0.02-0.33]
		3	85 (164/193)	0.16 [−0.02-0.33]
Item #3, “…*movements slowed down?”*	CAM 9, Psychomotor retardation	1	76.6 (147/192)	0.40 [0.25-0.54]
		2	76.0 (146/192)	0.43 [0.29-0.57]
		3	75.0 (144/192)	0.42 [0.28-0.56]

CAM: Confusion Assessment Method.

#### *Concurrent validity of* RADAR *compared with a DSM-IV-TR criterion defined delirium*

Table 5 presents the concurrent validity of RADAR items taken individually compared with a DSM-IV-TR criterion-defined delirium. Item #3 (*Were the patient’s movements slowed down?)* was the best performing item with a sensitivity of 65% and a specificity of 76%.

**Table 5:** **Concurrent validity of RADAR items compared with a DSM-IV-TR criterion-defined delirium**

RADAR items	Sensitivity % [95% CI]	Specificity % [95% CI]	Positive, predictive value, % [95% CI]	Negative, predictive value, % [95% CI]
Item #1: “*…was the patient drowsy?”*	26.1 [10.2-48.4]	94.1 [89.4-97.1]	37.5 [15.2-64.6]	90.4 [85.1-94.3]
Item #2: “*…trouble following your instructions*?”	21.7 [7.5-43.7]	86.5 [80.4-91.2]	17.9 [6.1-36.9]	89.1 [83.3-93.4]
Item #3: “…*movements slowed down?”*	65.2 [42.7-83.6]	76.3 [69.2-82.5]	27.3 [16.1-41.0]	94.2 [88.8-97.4]

CI: Confidence interval.

The concurrent validity of the RADAR scale (Table 6) was optimal among participants who underwent three to four RADAR screenings in the day (73% sensitivity with 67% specificity). Among this group (n = 113), 34 had a positive RADAR but no DSM-IV-TR delirium (positive predictive value of 19%). Low PPV of RADAR was a matter of concern for our team and so we undertook further analysis to determine the profile of these 34 participants who had positive RADAR but did not meet the DSM-IV-TR criteria for delirium. The analysis revealed that 100% of these individuals had acute cognitive impairment meeting the criteria set for CAM definite delirium (1 case), CAM probable delirium (24 cases), subsyndromal delirium (9 cases), definitions which were presented in an earlier paper [44-46].

**Table 6:** **Concurrent validity of RADAR compared with a DSM-IV-TR criterion-defined delirium according to the number of daily RADAR administrations**

Number of RADAR administrations (N)	Sensitivity % [95% CI]	Specificity % [95% CI]	Positive, predictive value, % [95% CI]	Negative, predictive value, % [95% CI]
1 to 4, (193)	65.2 [42.7-83.6]	71.2 [63.7-77.9]	23.4 [13.8-35.7]	93.8 [88.1-97.3]
1-2, (80)	58.3 [27.7-84.4]	77.9 [66.2-87.1]	31.8 [13.9-54.9]	91.4 [81.0-97.1]
3-4, (113)	72.7 [39.0-94.0]	66.7 [56.6-75.7]	19.0 [8.6-34.1]	95.8 [88.1-99.1]

CI: Confidence interval.

Since the number of RADAR administrations influences its sensitivity, we explored whether the number of RADAR items scored as positive was associated with detection of a DSM-TR-IV delirium. We found that each additional positive item of RADAR increased the odds of detecting a delirium by 43% (OR = 1.43; 95% CI = 1.16-1.77).

We also wanted to examine the relationship between the time available to complete a mental health assessment and a tool’s sensitivity. Based on our own dataset, we tested the efficiency of RADAR compared to our gold standard, i.e., the CAM and the HDS. In our study, the HDS took 20 minutes on average for completion and the CAM 10 minutes (total 30 minutes). We did three CAM assessments over a 7-hour period. When we took data from the three CAM assessments with the HDS for our efficiency analysis, we found a 100% sensitivity for DSM-IV-TR delirium. However, it took our research staff 50 minutes to obtain these data (Table 7). When we removed the data from one CAM assessment, sensitivity was reduced, as was the assessment time. Results showed that RADAR identified 73 times more cases of delirium per minute of interview than three interviews conducted with the CAM and the HDS. It should be remembered that CAM takes 10 minutes to administer and a cognitive test is required prior to its use [13].

**Table 7:** **Detection efficacy of RADAR (3–4 RADAR administrations)**

Number of daily CAM assessments	Time required (min)	Sensitivity	Specificity	Relative detection efficacy ^ a) ^
3 CAM + 1 HDS	30 + 20 = 50	100% (23/23)	95% (162/170)	1
2 CAM + 1 HDS	20 + 20 = 40	91% (21/23)	98% (167/170)	1.14
1 CAM + 1 HDS	10 + 20 = 30	78% (18/23)	98% (167/170)	1.30
RADAR	4 * 7 sec. = 0.5	73% (8/11)	67% (68/102)	73

CAM: Confusion Assessment Method; HDS: Hierarchic Dementia Scale.

^a)^The relative detection efficacy compares the number of successfully detected cases per minute using various assessments to our gold standard of 3 CAM interviews and one HDS.

Finally, we examined the concurrent validity of the RADAR scale in different sub-samples of participants (Table 8). Although the sensitivity of the tool remained practically the same for most of the groups, the specificity decreased among participants with cognitive impairment, psychomotor retardation and among the participants in the nursing home. In this last group, prevalence of delirium was low and for this reason, the sensitivity and specificity observed should be interpreted with caution.

**Table 8:** **Concurrent validity of RADAR compared with a DSM-IV-TR criterion-defined delirium in different sub-samples of participants (based on 3 to 4 RADAR administrations N = 113)**

Sub-samples of participants (n)	Sensitivity % [95% CI]	Specificity % [95% CI]	Positive, predictive value, % [95% CI]	Negative, predictive value, % [95% CI]
With cognitive impairment, (42)	71.4 [29.0-96.3]	42.9 [26.3-60.6]	20.0 [6.8-40.7]	88.2 [63.6-98.5]
Without cognitive impairment, (71)	75.0 [19.4-99.4]	79.1 [67.4-88.1]	17.6 [3.8-43.4]	98.1 [90.1-100.0]
With psychomotor retardation, (46)	70.0 [34.8-93.3]	44.4 [27.9-61.9]	25.9 [11.1-46.3]	84.2 [60.4-98.9]
Patients (hospital), (73)	70.0 [34.8-93.3]	81.0 [69.1-89.8]	36.8 [16.3-61.6]	94.4 [84.6-98.8]
Residents (nursing home), (40)	100.0 [2.5-100.0]	43.6 [27.8-60.4]	4.3 [0.1-21.9]	100.0 [80.5-100.0]

CI: Confidence interval.

### Perception of nursing staff concerning the use of RADAR in their clinical practice

The great majority of respondents (RN and LPN) found that (Table 9): 1) the items of RADAR were easy to understand (103 or 100%), 2) they had sufficient knowledge to be able to answer the items (102 or 99%), 3) the scheduled medication distribution process was a good time to carry out patient observation (102 or 100%) and, 4) completing RADAR did not result in an important increase in their workload (100 or 99%). Mean completion time for the RADAR scale was 7.2 seconds (SD = 4.4).

**Table 9:** **Feasibility and acceptability of RADAR by nursing staff (N = 103)**

Do you agree with the following statements? [Missing]	Agree n (%)	Disagree n (%)
The RADAR items are easy to understand.	103 (100.0)	
It’s easy to answer the RADAR items by observing the patient during the medication distribution process.	103 (100.0)	
I have sufficient knowledge to be able to answer the RADAR items.	102 (99.0)	1 (1.0)
I found the insertion of RADAR in the folder containing the medication distribution record convenient.	102 (99.9)	1 (1.0)
The medication distribution process is a good time to carry out patient observation.	102 (99.9)	1 (1.0)
The knowledge provided in the RADAR training package was sufficient for me to feel competent using the tool [2].	99 (98.0)	2 (2.0)
Completing the RADAR does not result in an important increase in my workload [2].	100 (99.0)	1 (1.0)

## Discussion

RADAR was developed with the aim of improving the recognition of delirium by addressing several limitations of current delirium tools, mostly related to their administration time, ease of use, generalizability and validity for clinical practice. The results of this study show that RADAR is a brief screening tool, one that nursing staff find easy to use, with psychometric properties supporting its use among older patients and residents with, or without, cognitive impairment. It was shown as well in Table 4 that the RADAR items can capture the hypoactive and hyperactive features of delirium.

It took nurses seven seconds on average to complete the 3-item RADAR; a definite improvement over the five to 15 minutes administration time that some of the existing delirium screening tools require. Less than one minute of nursing time per 24-hour period required to conduct four delirium assessments means that repeated assessments of delirium can be done without significantly increasing nurses’ burden. This is supported by the fact that 99% of nursing staff who completed the RADAR feasibility and acceptability questionnaire at the study end, stated that completing RADAR did not result in an important increase in their workload. As previously mentioned, the fluctuating nature of delirium throughout the day means frequent mental health assessments should be an essential feature of any screening instrument. But, to ensure frequency, the instrument should only take a short time to administer. This study showed that the probability of getting a positive RADAR increases with the number of daily RADAR screenings (11% of RADAR positive among participants with one RADAR screening per day vs. 40% for those with four per day).This finding reinforces the need for frequent daily screening of delirium and RADAR with its 7 seconds administration time and its approval rating from clinical staff, is certainly a new option to consider for clinical practice.

The several strategies put forward to maximize ease-of-use of RADAR by nursing staff appear to have been successful since all RN and LPN who completed the RADAR feasibility and acceptability questionnaire reported that RADAR items were easy to understand and easy to rate by observing the patient during the medication distribution process. Participating staff also found the insertion of the RADAR scale into the folder containing the medication administration records was practical. It should be noted that throughout all four steps leading to the final version of RADAR, the researchers consulted nurses to obtain their input about RADAR, in particular on how easy the scale was to use in their clinical settings. The RADAR training program in its present form is an example of improvements that emerged from those discussions. Because of its short duration (less than 15 minutes excluding bedside coaching sessions) the training package of instructions, videos with case-based scenarios and one-on-one bedside coaching sessions, is quite easy to implement, even in fast-paced clinical settings (see www.fsi.ulaval.ca/RADAR). The results of our study support the use of multi-faceted training since 98% of respondents found the information given during the RADAR training was sufficient for them to feel confident using the tool. In addition, the high percentage of agreement observed between the RADAR administered by the RA and that administered by beside nurses, indicates that the training given succeeded in conveying clear instructions about the proper use of the tool. The efficacy of multi-faceted training to improve nurses’ knowledge about delirium and its recognition has also been demonstrated in the literature [47].

The sensitivity and specificity values shown by the RADAR are in accordance with the general expectations of a screening tool: achieving fairly high sensitivity while guaranteeing appropriate clinical specificity. As expected, given the fluctuating nature of delirium that necessitates repeated assessments throughout the day, we observed the highest sensitivity among participants whose RADAR score was based on 3–4 daily RADAR screenings. Among this particular group, RADAR was found to be sensitive (73%) and moderately specific (67%) for a delirium according to the DSM-IV-TR criteria. It should be noted that RADAR took 7 seconds on average and more than 50% of the time RADAR was completed by LPN. These results compare relatively well with the performance of other delirium tools. For example, although the CAM has been shown sensitive (94% to 100%) and specific (90% to 95%) for delirium in validation studies [14], lower sensibilities (19% to 47%) were obtained in studies that approximated actual clinical practice [4,6,8,13]. In routine clinical practice, conditions may not always be optimal for screening (e.g. lack of proper training in the use of the CAM, CAM ratings done with extremely brief cognitive assessment, or the presence of a large population with dementia) [13]. RADAR can be used as a screening scale for delirium. Or, RADAR could also screen individuals for signs of delirium in order to refer them for more in-depth delirium testing when a positive score is obtained; a method suggested by other researchers [32]. With this method, the clinical implication for the false-positive in the context of delirium screening has no serious consequences on the nursing staff workload. Administering a more comprehensive delirium tool to the false-positive patients is still more time-efficient than conducting systematic screening of all patients using this same tool. It is important not to forget that only 18% of all administration of RADAR ended with a positive. It is also worth noting that further analysis revealed all the false-positive participants (34 participants with a positive RADAR but no DSM-IV-TR delirium) were meeting other criteria sets used in the literature for delirium or subsyndromal delirium [44,48-51].

To address a limitation of existing delirium screening tools with regards to their generalizability, we included patients and residents, both with and without dementia in this validation study. Due to the relatively small number of participants in each group, in-depth analysis of the performance of RADAR among these different subgroups was not possible. However, our results showed that nursing staff in both acute and long-term care settings found the RADAR easy to use and this despite a 21% prevalence of dementia in the overall study sample.

This study has several strengths. First, delirium and its symptoms were measured using an instrument recognized for its psychometric properties (i.e., the CAM). Second, ratings of delirium with the CAM were based on a 7-hour observation period as well as additional sources of information (staff and family members, medical chart review and a baseline cognitive assessment). Third, in order to minimize contamination bias, the research assistant responsible for collecting data related to RADAR was blinded to the delirium status of patients/residents.

The study findings should be interpreted in light of the following limitations. Despite the high recruitment rate of nursing staff, 25% did not complete the RADAR feasibility and acceptability questionnaire at study end, which may have introduced a non-response bias. However, except for three participants who decided to withdraw their consent (out of 139 participants), participating staff’s reasons as to why questionnaires were not completed as planned (maternity, vacation, sick leave, transfer, end of contract), were unrelated to any factors that could have negatively affected their perception of the tool. For this reason, we believe the likelihood of a non-response bias is minimal. Another limitation of this study relates to the DSM criteria used to define delirium. First, one of the requisite criteria of the DSM-IV-TR edition is the presence of an alleged medical cause of delirium. Identifying a specific medical cause of delirium among older patients/residents can be quite difficult, given the high level of comorbidity among this population and so was not possible in this study. Exclusion of this criterion may have inflated the prevalence rates for delirium. Besides, since the start of this study, a newer version of the DSM has been published (DSM-5) in which changes were made regarding the criteria set for delirium. We did further analysis applying these criteria to our sample and we ended up having the same number of cases of delirium with the exact same participants. In other words, based on our sample, the application of the DSM-IV-TR versus the DSM-V criteria does not change the results in Table 6. Finally, observation of the patient during distribution of medication is necessary to score the RADAR. If a patient does not take any medication during hospitalisation, using the RADAR is of limited value. Future study should test the validity of the RADAR when administered following other clinical activities.

## Conclusion

Since RADAR is efficient, reliable, sensitive and very well accepted by nursing staff, it can be viewed as an appropriate new option for delirium screening among older adults with, or without cognitive impairment, in both hospitals and nursing homes. Other projects are now underway to validate the RADAR among another population (e.g., middle-aged adults) as well as in other clinical settings, such as home care, emergency department, medical intensive care unit, and palliative care.

## Additional files

Additional file 1:
**Recognizing acute delirium as part of your Routine.**

Additional file 2:
**Steps leading to the final version of RADAR.**

## Abbreviations

CAM: Confusion assessment method
DSM: Diagnostic and statistical manual of mental disorders
LPN: Licenced practical nurse
RN: Registered nurse
CPNP: Candidate to the profession of nursing practice
RA: Research assistant
HDS: Hierarchic dementia scale
SMAF: Functional autonomy measurement system
CCI: Charlson comorbidity index
SE: Sensitivity
SP: Specificity
PPV: Positive predictive value
NPV: Negative predictive value
M: Mean
SD: Standard deviation
CI: Confidence interval
