# Supplementary figures and images for: Recognizing acute delirium as part of your routine [RADAR]: a validation study
Source: BMC Nurs. 2015 Apr 1;14:19. doi: 10.1186/s12912-015-0070-1 (PMC4384313; doi:10.1186/s12912-015-0070-1)

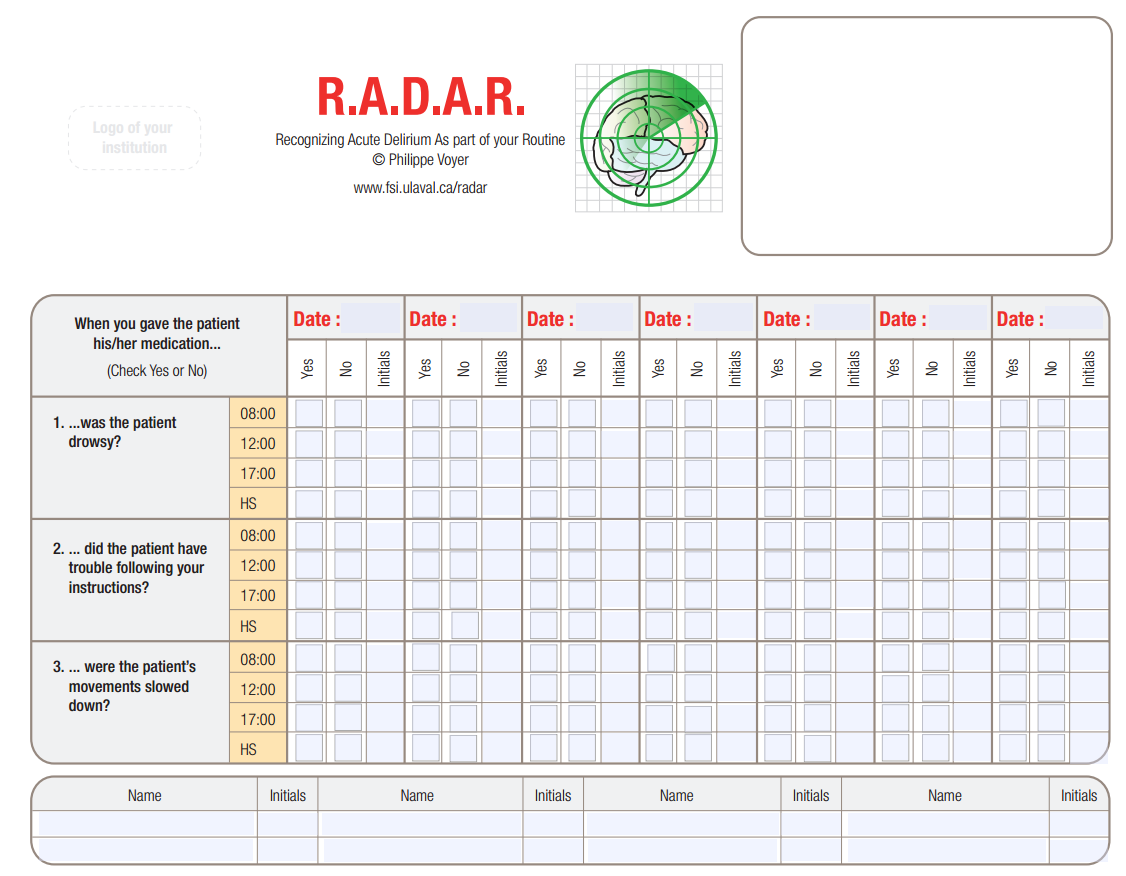

Supplement: Additional file 1: — Recognizing acute delirium as part of your Routine. [file 12912_2015_70_MOESM1_ESM.docx]
