# Supplementary material for: Recognizing acute delirium as part of your routine [RADAR]: a validation study
Source: BMC Nurs. 2015 Apr 1;14:19. doi: 10.1186/s12912-015-0070-1 (PMC4384313; doi:10.1186/s12912-015-0070-1)
Supplement: Additional file 2: — Steps leading to the final version of RADAR. [file 12912_2015_70_MOESM2_ESM.docx]

| STEPS LEADING TO THE FINAL VERSION OF RADAR |
| --- |
| STEP 1  Development and pre-testing of RADAR items |
| For each of these symptoms of delirium specified in the DSM-III-TR and DSM-IV-TR (acute onset, fluctuation, inattention, disorganized thinking, altered level of consciousness, disorientation, memory impairment, perceptual disturbances, psychomotor agitation or retardation), researchers developed several “yes-no” questions to identify the presence of signs that could be indicative of the symptoms; signs that nurses could observe during the medication distribution process. The resulting 45 questions were then tested for clarity by nursing staff (19 registered nurses, and 29 licensed practical nurses) in one acute care hospital and one long-term care facility, both located in Quebec City. For each question, nurses had to indicate if the question was clear and easy to understand. In addition, from among the proposed questions for each delirium symptom, they were to indicate their top three preferences. Researchers then selected the best rated question per symptom to form the 12-item RADAR. |
| STEP 2  Evaluation of content validity of RADAR (12 items) by delirium experts |
| A group of five experts in the field of delirium (1 psychogeriatrician, 2 psychiatrists and 2 geriatric clinical nurse specialists) were asked to rate the content validity of each item on a 5-point Likert scale. For each item, they were asked: “Is the item (observable behaviour) an adequate indicator of the targeted delirium symptom?” The experts were also to evaluate if the 12 items of RADAR represented all delirium symptoms described in the DSM-IV-TR. Following this first consultation, researchers modified two items and subsequently resubmitted them to the five experts. In the second round, all 5 experts rated all items of RADAR as valid and they stated that RADAR captures all the symptoms of delirium categories recommended in the DSM-IV-TR. |
| STEP 3 - Pilot study  Feasibility study and preliminary data on the 12-item RADAR |
| Objectives: To test the methodology to be used in the large scale RADAR study and to obtain preliminary data on RADAR (inter-rater reliability, concurrent validity against DSM-IV-TR criteria for delirium, ease-of use by nursing staff and training package quality).  Settings: One acute care hospital and one long-term care setting, both located in Quebec City.  Participants: four registered nurses and 19 licenced practical nurses; 29 patients and 28 residents.  Methods: Participating nurses received a 15-minutes PowerPoint presentation on delirium and on the use of RADAR. They were to complete RADAR each time they distributed medication to patients/residents who had given their informed consent. To obtain data for testing the inter-rater reliability, a research assistant was to administer RADAR at the same time as the bedside nurse. A second research assistant to assess the presence of delirium symptoms using the Confusion Assessment Method (CAM). The concurrent validity of RADAR items against the DSM-IV-TR criteria of delirium was explored. A questionnaire given to nursing staff gauged the ease of use of the scale and evaluated the training they received.  Results and adjustments for the large scale study (Step 4): This pilot study showed that the methodological approach proposed was feasible and therefore could be replicated in a large scale study. Based on the results obtained, the research team members agreed to two modifications. First to attain their goal of improving the inter-rater reliability of the RADAR items which varied from 73% to 100%, researchers enhanced the RADAR educational package by providing multifaceted training. In addition to the instruction in the PowerPoint presentation, training videos including case-based scenarios were added. Second, in an attempt to keep the RADAR administration time to a minimum, seven items were dropped, due either to their low prevalence in the study sample or a low sensitivity with delirium. RADAR was well received by most of the participating nursing staff: 95% or more found that: 1) the items of RADAR were easy to understand; 2) they had sufficient knowledge to be able to answer the items and, 3) the scheduled distribution of medication was a good time to carry out patient observation. As well, 22% found that completing RADAR increased their workload considerably. |
| STEP 4 - Large scale study  Evaluation of the 5-item RADAR scale |
| Objectives: Evaluation of the psychometric properties and ease of use of the 5-item RADAR.  Settings: Three medical units of an acute care hospital and five units in a long-term care facility in Quebec City.  Participants: 40 registered nurses and 63 licenced practical nurses; 140 patients and 52 residents.  Methods: Same as pilot study  Results and Adjustments for Step 5: Percentages of agreement between the RADAR items administered by the bedside nurse and the RA varied from 83% to 100%. The combination of the three best items yielded a sensitivity of 67% and a specificity of 58% against a DSM-IV-TR. The majority of nursing staff who completed the end-of-study questionnaire (n = 84) found that: 1) the items of RADAR were easy to understand (96%); 2) they had sufficient knowledge to be able to answer the items (99%); 3) the medication distribution process was a good time to carry out patient observation (94%) and, 4) completing RADAR did not result in an important increase in their workload (96%). The mean time to complete the 5-item RADAR was 15.1 seconds (SD = 6.5). Based on the results obtained, the two items with the lowest sensitivities (13%) were dropped and the RADAR training was upgraded by adding bedside coaching sessions as well as a review of RADAR items every 4 weeks (or as needed). |
